# Co-design of a systems-wide approach (CONNECTS-Food) to promote adoption of whole-school approaches to food

**DOI:** 10.1017/S1368980025101353

**Published:** 2025-10-17

**Authors:** Wendy Burton, Niamh O’Kane, Jayne Woodside, Charlotte E.L. Evans, Harry Rutter, Suzanne Spence, Sara M. Ahern, Amir Sharif, Tim Baker, Maria Bryant

**Affiliations:** 1 Department of Health Sciences, University of York, Heslington, York YO10 5DD, UK; 2 Centre for Public Health, Queen’s University Belfast, Institute of Clinical Sciences A, Grosvenor Road, Belfast BT12 6BJ, UK; 3 School of Food Science and Nutrition, University of Leeds, Woodhouse, Leeds LS2 9JT, UK; 4 Department of Social & Policy Sciences, University of Bath, Claverton Down, Bath BA2 7AY, UK; 5 Human Nutrition and Exercise Research Centre, Population Health Sciences Institute, Newcastle University, Newcastle upon Tyne NE1 7RU, UK; 6 Better Start Innovation Hub, Bradford Institute of Health Research, Bradford Royal Infirmary, Duckworth Lane, Bradford BD9 6RJ, UK; 7 Faculty of Management, Law and Social Sciences, University of Bradford, Richmond Road, Bradford BD7 1DP, UK; 8 Hull York Medical School, University of York, Heslington, York YO10 5DD, UK

**Keywords:** School food system, Action Scales Model, Whole-school approach to food, Co-design, Systems-wide approach

## Abstract

**Objective:**

To co-design a systems approach aimed at promoting the wide-scale adoption of whole-school approaches to food in UK primary schools to improve school food environments, food provision and dietary intake in children.

**Design:**

A systems framework (Action Scales Model) was used to guide the co-design of the systems approach. The process involved identifying leverage points within the UK primary school food system that, if influenced, could alter the way in which the system functions. Actions were then agreed upon to influence those leverage points.

**Setting:**

Co-design workshops were held online between September 2021 and February 2022.

**Participants:**

Members of the co-design team comprised twelve school stakeholders (headteachers, school food improvement officers, catering leads, representatives of UK school food organisations and a dietician) and a team of researchers with expertise in school food, systems thinking and intervention development. Our partnership board included decision-makers and advocates of the whole-school approach to food in England and Northern Ireland.

**Results:**

Identified leverage points included the priorities of headteachers, who are instrumental in instigating whole-school approach to food adoption. Direction from local and national policymakers was also identified. Actions to influence these leverage points included providing direct support to schools (through an online resource) and encouraging policymakers to monitor the adoption of the approach.

**Conclusion:**

The methods described here can be replicated by others to promote the adoption of whole-school approaches to food in other contexts and contribute to the growing literature on developing systems-wide approaches to promote the adoption of public health initiatives.

Schools are a key setting to promote the health and well-being of children and adolescents^(1)^. Primary schools, where children attend from the ages of 5–11 years, have been highlighted as important, as this is a key time within the life course where attitudes and behaviours towards health are shaped, which are known to track into adulthood^(2,3)^. The role of schools in promoting child health has been emphasised by the WHO since 1986^(4–6)^. A key aspect of this is a ‘whole-school approach to food’, a settings approach that includes consideration of the quality of all food available to children during the school day, the extent to which children are given the opportunity to learn with and about food and a school’s cultural relationship with food^(5)^. The reach and potential impact of such an approach are substantial due to the role that schools play in supporting children who live in disadvantaged circumstances and because children consume approximately one-third of their food intake in schools during the school day^(7)^. Although the evidence is still developing, a Cochrane systematic review and meta-analysis undertaken in 2015^(8)^ reported that whole-school approaches to food have positive effects on BMI and fruit and vegetable intake. However, in the UK, there is a lack of consistency in whole-school approaches to food adoption, both locally and nationally, resulting in inequitable access to its benefits^(9)^.

Schools are considered unique complex systems, with multiple competing demands and a diverse range of actors including senior leadership teams, governors, teachers and parents^(10)^. The diversity between school systems is broad, with each operating within its own context and possessing its own components, structures, rules and feedback loops^(11)^. Schools sit within broader political, health and food systems^(10,12)^. Hence, a systems-wide approach is needed to promote the wide-scale adoption of whole-school approaches to food.

The role of systems approaches in supporting the adoption and implementation of public health initiatives has been developing over the last two decades^(13)^. To support this, a range of frameworks and models have been developed (e.g. the Intervention Level Framework^(14)^, Action Scales Model^(15)^ and the Public Health 12 Framework)^(16)^ that involve the identification of leverage points, which are places within a system that can generate change if influenced. It has been proposed that influencing multiple leverage points across multiple parts of a system offers the greatest potential^(17)^, as well as the meaningful involvement of stakeholders using a participatory approach (e.g. co-design), to identify leverage points and agree upon a set of actions that are most likely to result in systems-wide change^(15,18)^. However, before a systems-wide approach can be developed, an understanding of how a system functions, through methods such as systems dynamic modelling, network analysis or group model building, is needed^(19)^.

Currently, there is no consistent definition of what a whole-school approach to food means in practice, and school-level interventions often focus on a particular feature (e.g. school policy, food environment or fruit and vegetable provision) rather than the approach as a whole^(8)^. There is also a lack of initiatives aimed at promoting wide-scale adoption. There is, however, growing interest in the broader school system (beyond immediate school settings) and the role it plays in the adoption and implementation of public health initiatives in schools. For example, the work of Langille and Rogers^(20)^ and McIsaac *et al*.^(21)^ explored systems-level factors that influence the adoption of school food and physical activity initiatives in Canada, highlighting the role of national policy and the priority placed on academic achievement. But to date, this understanding has not been used to inform a systems-wide approach aimed at promoting the adoption of school food initiatives. In a previous study, we developed a map of the UK primary school food system using a group model-building approach^(22)^, which identified four domains of influence on children’s dietary intake during the school day: leadership, culture and curriculum; child food choice; school food offer; and home environment. This paper describes the co-design of a systems-wide action plan, using our map of the UK primary school system to promote wide-scale adoption of whole-school approaches to food, thus improving school food environments, school food provision and dietary intake in children (both within and outside of school). Specifically, our study had three aims: (1) to define what a whole-school approach to food means in practice, (2) identify leverage points from within the school food system that influence the adoption of whole-school approaches to food and (3) agree upon a set of actions to influence these leverage points.

## Methods

### Theoretical approach

#### Action Scales Model

The Action Scales Model (ASM)^(15)^ was used as a guide to develop the action plan, which conceptualises potential leverage points within a system as weights and a set of scales; the largest of the weight depicts the ‘beliefs’ or paradigm underpinning a system, which, if influenced, offer the greatest opportunity to reshape the ways in which that system functions. In contrast, the smallest of the weights depicts ‘events’ within a system (e.g. one-off training events), which offer a quick fix and are often the easiest to implement but generate little leverage for systems change. The practical use of the ASM includes convening a team of stakeholders to identify leverage points within a system and then categorising them by weight category. Actions to influence each leverage point are then agreed upon, with the aim of including a range of actions across each weight category. The agreed-upon actions are represented in an action plan (see Table 1, for example).

**Table 1.** Example leverage points and actions according to each weight category of the Action Scales Model^(15)^

Proposed area(s) of the school food system that influences the whole-school approach to food adoption^(22)^	Leverage point	Degree of leverage within overall system (according to Action Scales Model) (*highest to lowest; beliefs of system architects, system goals, system structures, system events)*	Example action
Priorities of headteachers and senior leadership teams	Extent to which school headteachers believe in the value of a whole-school approach to food	Belief	School food organisation provides scientific evidence to headteachers on how the approach benefits children
Extent of DfE/Ofsted monitoring, local authority buy-in with whole-school approach to food, priorities of school governors	Extent to which schools are required to work towards the delivery of a whole-school approach to food	Goals	Local authorities monitor the adoption of the approach in their region
Priorities and skills of teachers, extent of DfE/Ofsted monitoring, priorities of headteachers	Extent to which school staff take responsibility for delivering a whole-school approach to food	Structures	Headteacher enlists a whole-school approach to food champion to lead delivery of the approach
Priorities and skills of teachers, awareness of initiatives and resources, priorities of headteachers and senior leadership teams	Extent to which teachers understand how to introduce food into the wider curriculum	Events	Headteachers provide training to school staff on how to incorporate food into the wider curriculum

DfE, UK Government Department for Education; Ofsted, Office for Standards in Education, Children’s Services and Skills.

### CONNECTS-Food action plan development process

**Figure 1.**
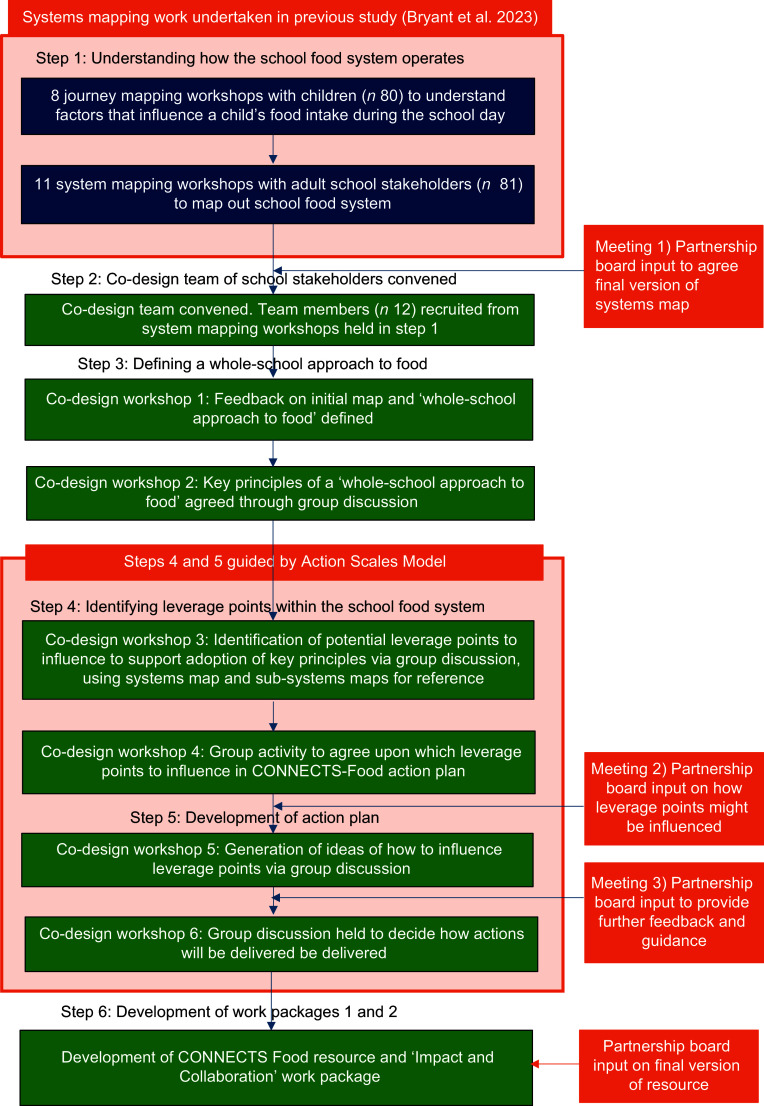
Co-design process.

A co-design method was used to develop the CONNECTS-Food action plan. The process involved six steps (Figure 1): (1) gaining an understanding of how the school food system operates (mapping the school food system); (2) convening a co-design team of school stakeholders; (3) defining a whole-school approach to food; (4) identifying leverage points within the school food system, which, if influenced, could support adoption of whole-school approaches to food; (5) identifying and agreeing upon actions to influence those leverage points; and (6) development of two work packages to support schools to implement the approach. Step 1 was undertaken in a previous study^(22)^. Steps 2–6 are part of the present study. Our work was supported by a partnership board comprising decision-makers and advocates of the whole-school approach to food in England and Northern Ireland. The content of co-design workshops and partnership board meetings is summarised here, with full details provided in Additional File 1.

### Partnership board

The partnership board (*n* 10) met four times during the study and included representatives from Public Health England, the Public Health Agency in Northern Ireland, the UK Government Department for Education (DfE), the Education Authority in Northern Ireland, GENIUS network (network of school food advocates), UK-based organisations aiming to promote children’s access to healthy food at school (School Food Matters^(23)^ and Sustain)^(24)^ and representatives from three local government authorities in England.

### Step 1: Understanding how the school food system operates

We have previously reported on how we developed a map of the school food system using a group model-building approach to understand how the system operates^(22)^; therefore, the process is only described briefly here.

#### Journey mapping workshops with children

Eight in-person ‘journey mapping’^(25)^ workshops were held with primary school children (*n* 80) aged 5–11 years in eight schools across four regions of the UK (Belfast, Bradford, Leeds and Newcastle) to understand factors within the school food system that influence a child’s food intake during the school day. During workshops, researchers performed an activity where each child picked a card denoting a particular moment in the day (e.g. morning break) and the child described what normally happened at that time. During the activity, researchers mapped out the children’s school day ‘journey’ against an image of a timeline on a white board, which was reviewed at the end to facilitate further discussion.

#### Systems mapping workshops with adult school stakeholders

A series of systems mapping workshops with adult school stakeholders was held online via Zoom in conjunction with the journey mapping workshops. Systems mapping workshops were attended by eighty-one school stakeholders (headteachers, governors, parents, teachers, caterers and representatives of national school food organisations) who each attended one workshop. Some stakeholders were based in the same four regions of the UK as the journey mapping workshops, while others, representing national organisations, came from other parts of the UK. The journey maps produced by the children were presented during the workshops to facilitate discussions around the factors that influence child food intake throughout the school day. The series of workshops resulted in an initial map of the school food system.

Partnership board meeting 1: Following the development of the initial systems map, a partnership board meeting was held. An image of the initial systems map was presented to the board, and the partnership board was invited to ask questions and suggest if any nodes or connections were missing.

### Step 2: Convening of co-design team

We convened a co-design team of twelve school stakeholders to develop the CONNECTS-Food action plan alongside a team of researchers with expertise in school food, systems thinking and intervention development. All stakeholders invited to be part of the co-design team (*n* 81) had attended a systems mapping workshop that was held in step 1, whereby stakeholders were informed of the opportunity to join the co-design team at the end of each workshop, with an email sent around after the workshop to provide further details of what would be involved^(22)^. There was no limit on the number of stakeholders permitted to join the group and no eligibility criteria, although we aimed to include a range of stakeholders to represent a variety of perspectives. Co-design workshops lasted 2 h and were held online using Zoom, as favoured by the co-design team. All workshops were recorded so that discussions could be revisited afterwards to ensure nothing was missed. Six workshops were held over 6 months between September 2021 and February 2022.

### Step 3: Defining a whole-school approach to food (study aim 1)

#### Co-design workshop 1

In the first co-design meeting, the team provided feedback on the initial systems map developed in step 1 to enable the final version to be developed. In the second half of the workshop, the co-design team was asked to define what a whole-school approach to food means in practice by listing objectives of the approach (e.g. to make lunchtimes a vital element of school life), as well as detailing who was expected to adopt each objective (e.g. headteachers). After the meeting, three members of the research team (WB, JW, NOK) further expanded the list of objectives by reviewing publicly available resources designed to support schools in implementing a whole-school approach to food. Listed objectives were thematically grouped (by WB and NOK) to set out key themes/principles that underpin the approach.

#### Co-design workshop 2

In workshop 2, each key principle, as proposed by the research team, was presented to the co-design team. Co-design team members were asked to refine concepts and reach agreement on the final set of principles.

### Step 4: Identifying leverage points within the school food system (study aim 2)

#### Co-design workshop 3

**Figure 2.**
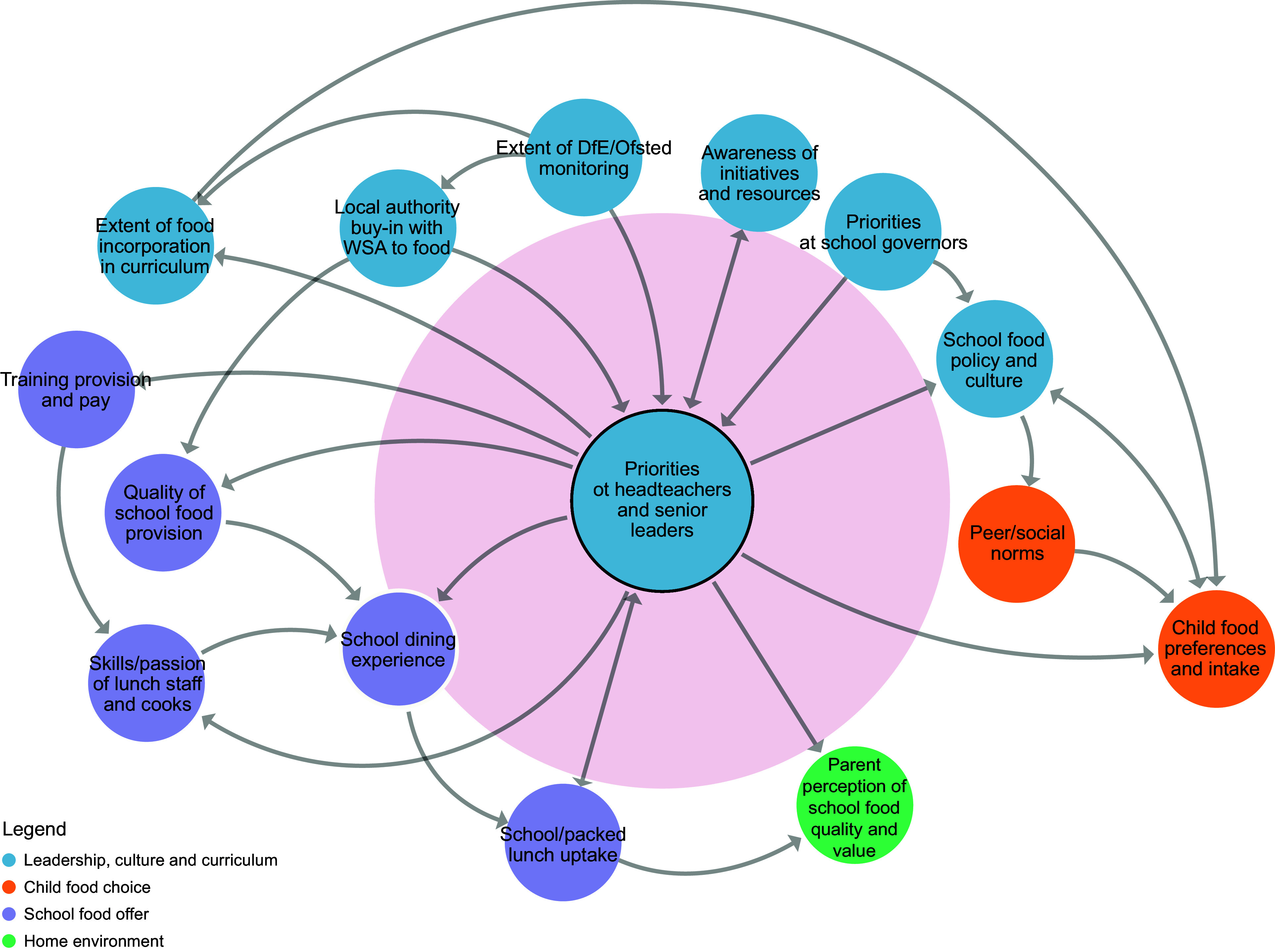
Sub-system 1: Priorities of school leadership teams.

**Figure 3.**
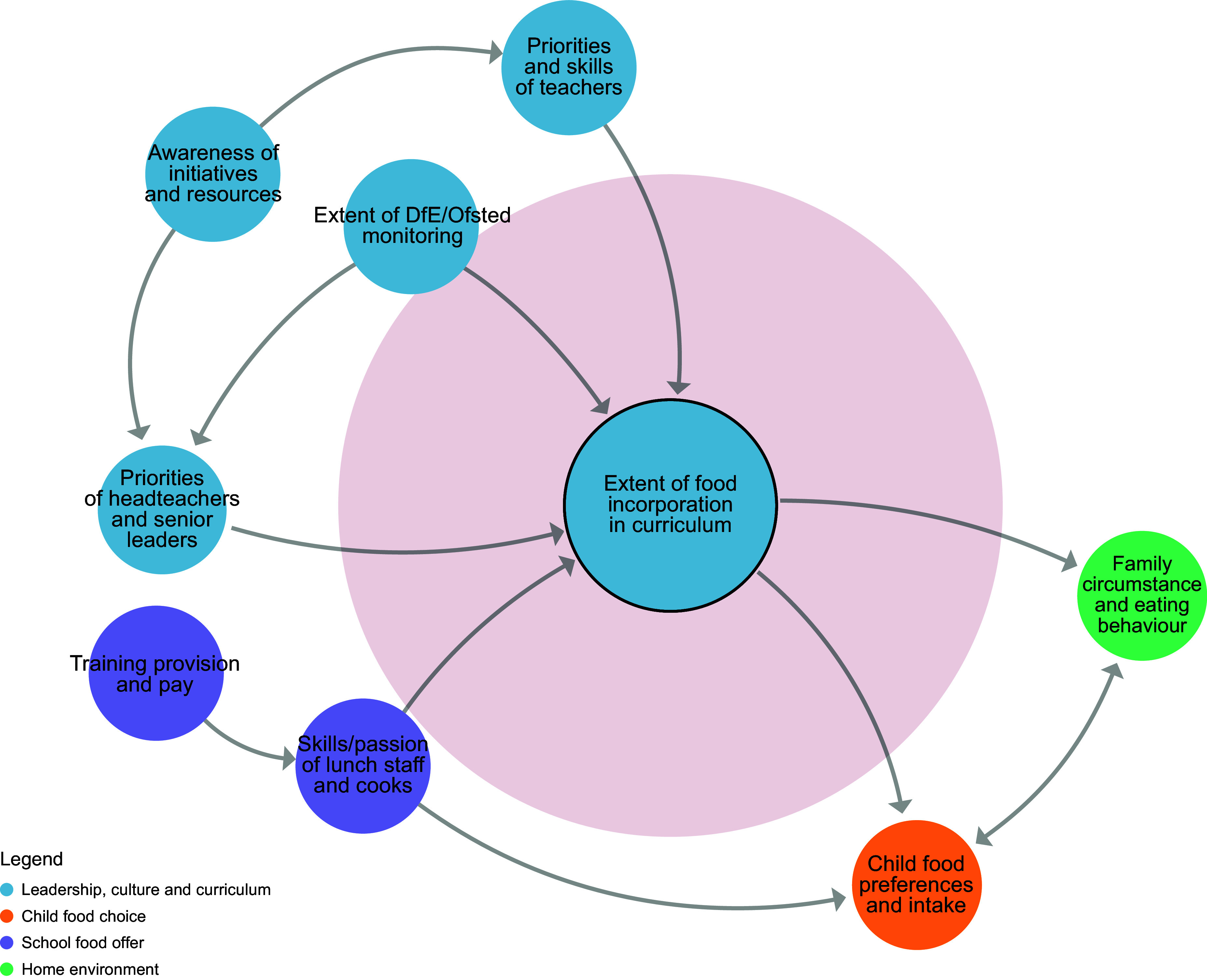
Sub-system 2: Food on the curriculum.

**Figure 4.**
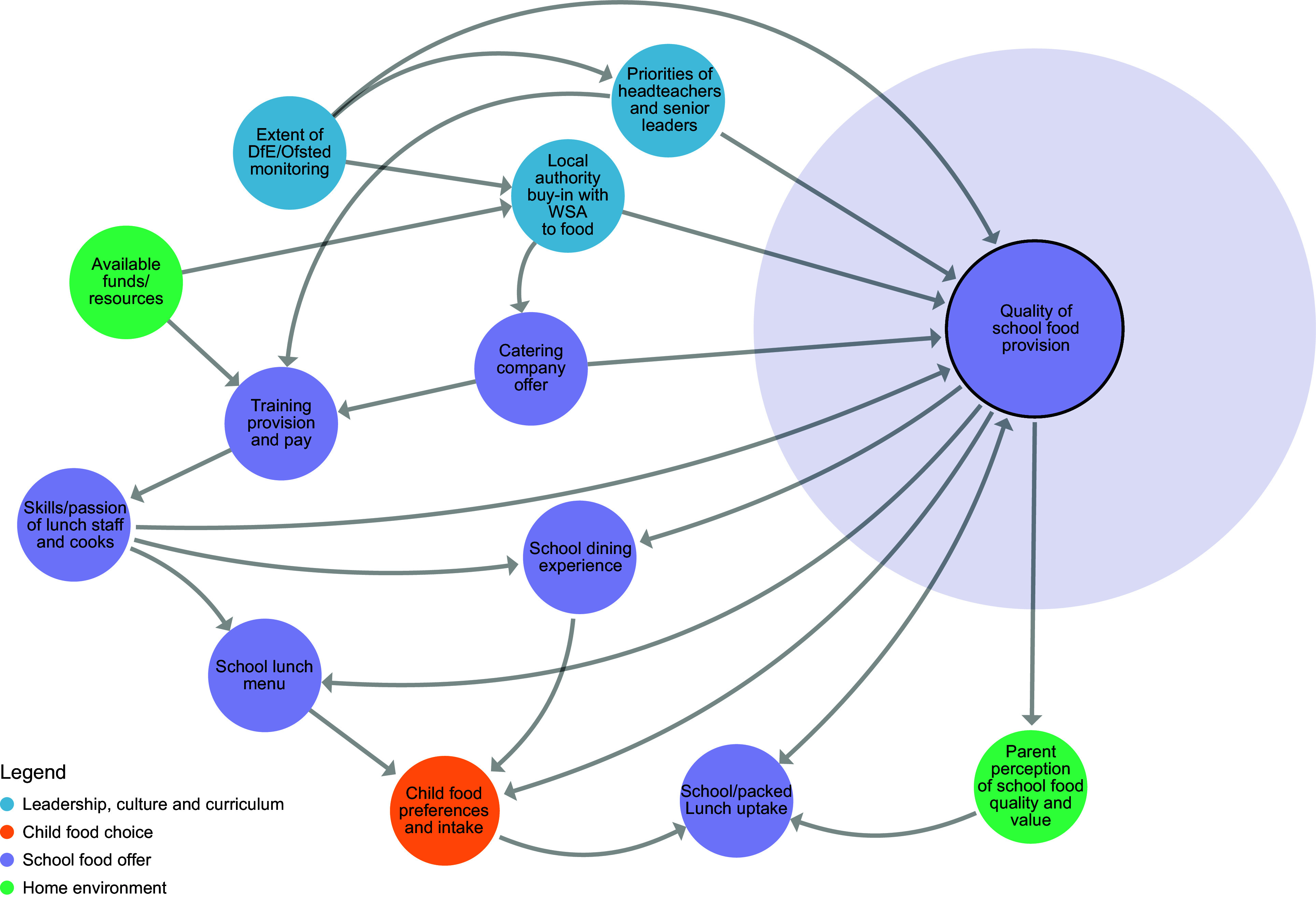
Sub-system 3: School food provision.

**Figure 5.**
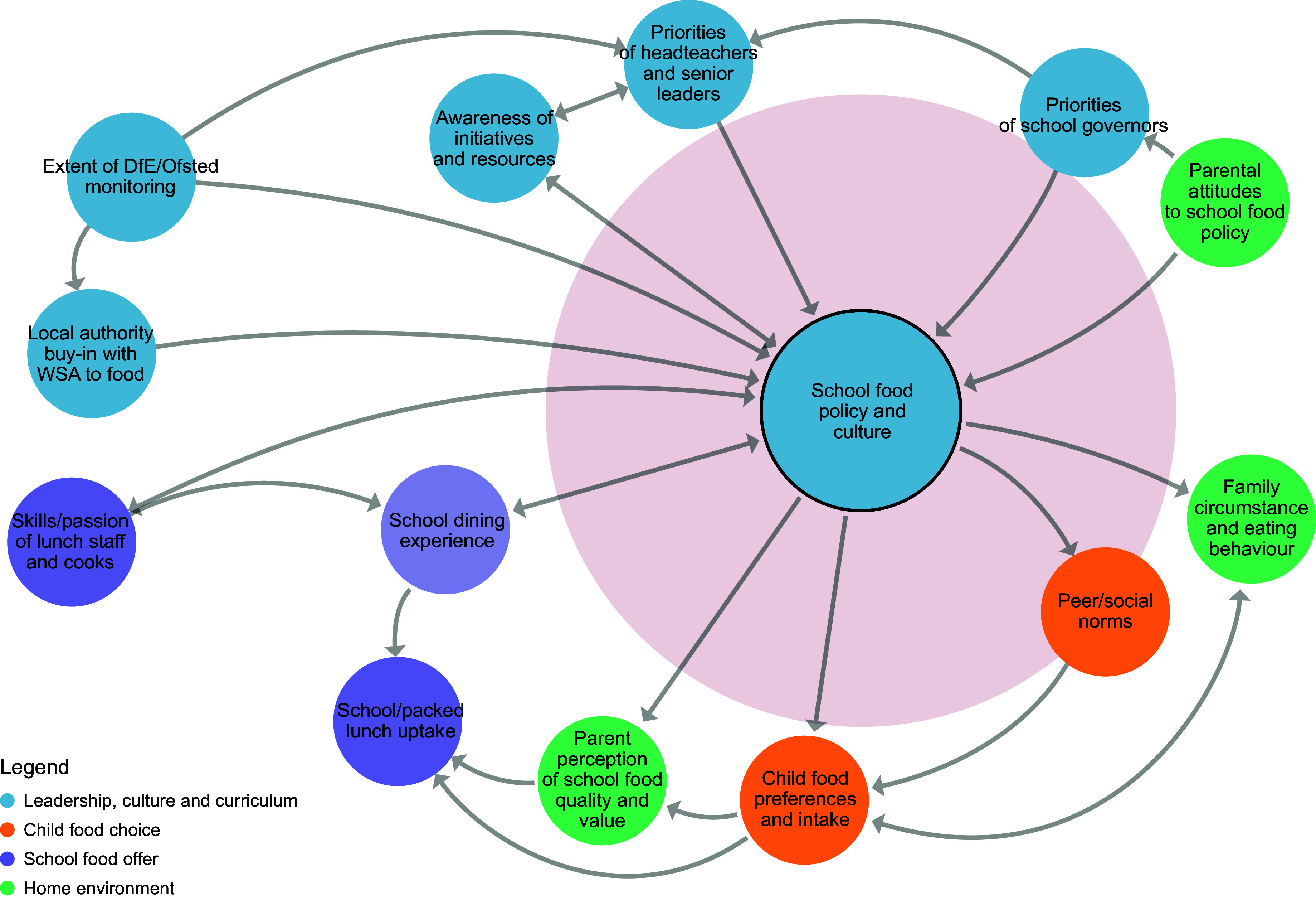
Sub-system 4: School food policy and culture.

**Figure 6.**
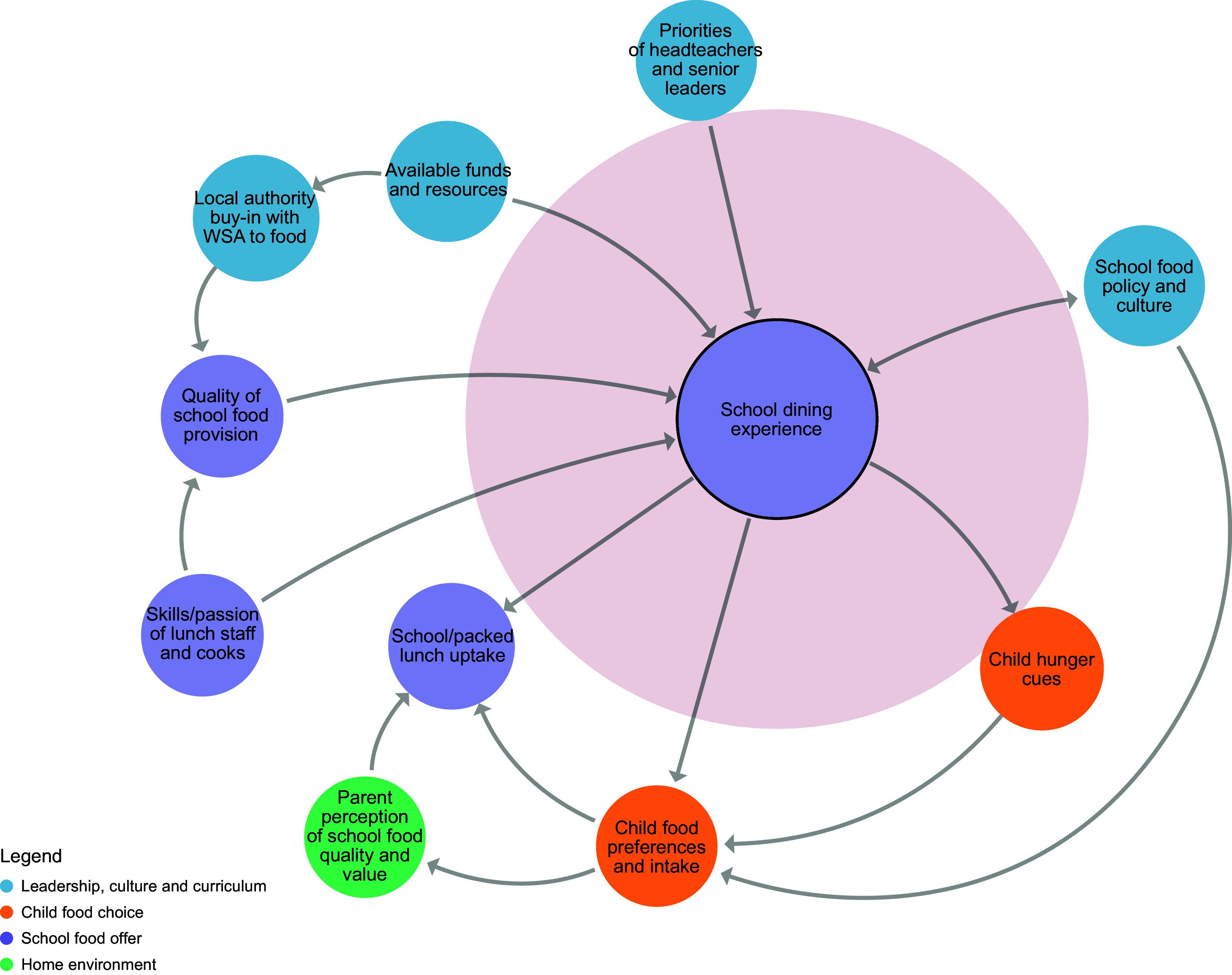
Sub-system 5: School dining experience.

**Figure 7.**
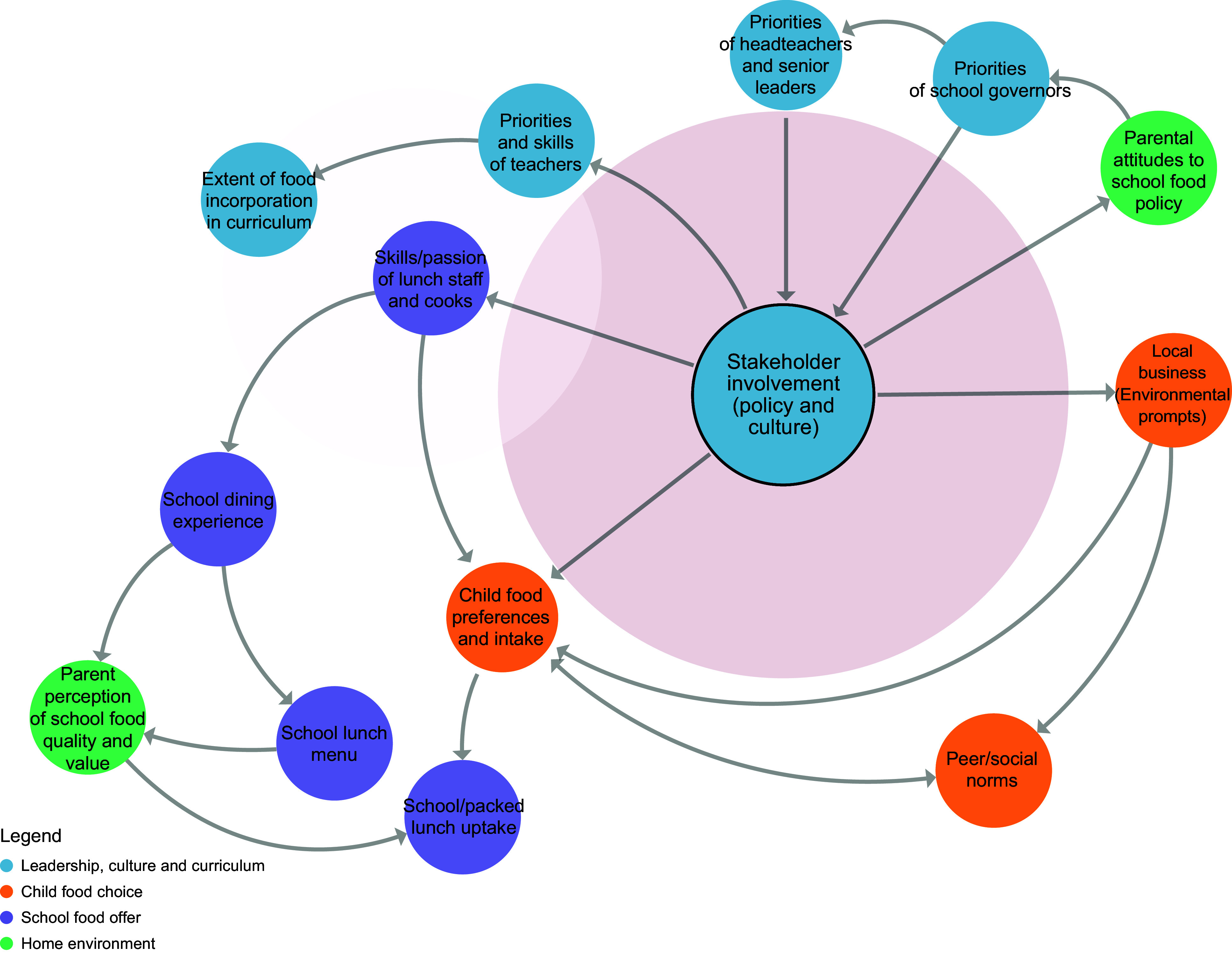
Sub-system 6: Stakeholder involvement.

**Figure 8.**
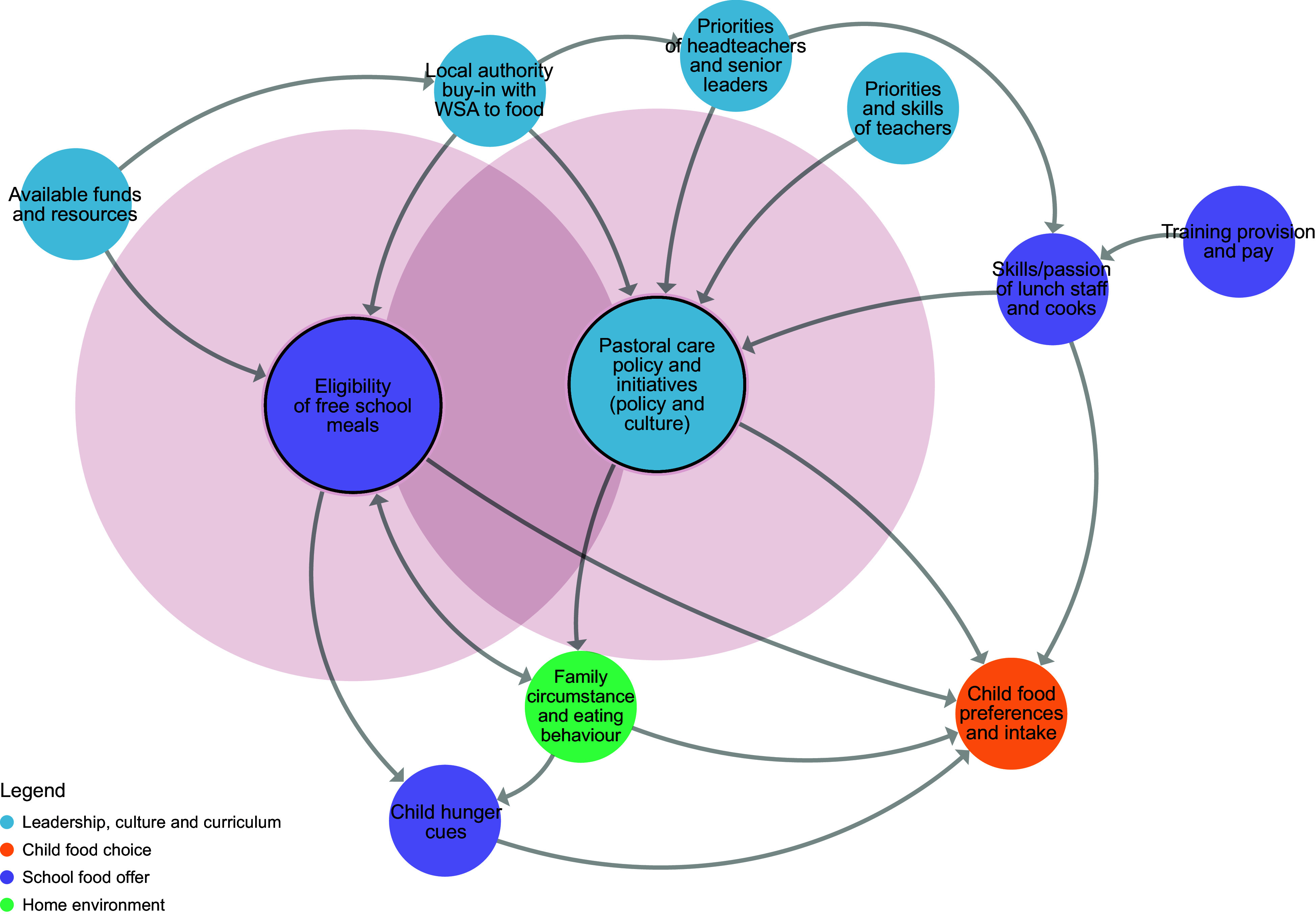
Sub-system 7: Pastoral care.

Prior to workshop 3, a member of the research team considered each key principle within the context of the school food system map and proposed which factors within the system would influence the adoption of each principle. A series of sub-systems maps was then developed using Kumu systems mapping software^(26)^, one for each principle (Figures 2–8). During the workshop, the group considered each sub-system in turn to identify potential leverage points to influence the adoption of each key principle.

#### Co-design workshop 4

During workshop 4, leverage points to influence as part of the CONNECTS-Food action plan were agreed by considering their feasibility to influence and potential systems-wide impact (as guided by ASM weight categories). This was achieved by undertaking a ranking activity as described in Additional File 1.

### Step 5: Development of the action plan (study aim 3)

#### Partnership board meeting 2

Following agreement by the co-design team on which leverage points to influence, a meeting was convened with the partnership board for feedback and guidance. Specifically, the partnership board members were asked to draw on their experience and expertise from a regional and national perspective to generate ideas on how they thought the leverage points could be influenced. During the meeting, an initial draft of the action plan was developed.

#### Co-design workshop 5

Co-design team members were asked to develop the initial action plan further by building upon the actions proposed by the partnership board, as well as considering additional actions. The team was also asked to consider whether they were aware of work being undertaken locally to influence the adoption of whole-school approaches to food to avoid replication or consider whether CONNECTS-Food actions might support this work.

#### Partnership board meeting 3

After the co-design team had developed the action plan further, the partnership board convened again to provide further feedback and guidance. The partnership board was also asked if they were involved in or aware of work at a national level aimed at influencing the adoption of whole-school approaches to food to understand whether the CONNECTS-Food action plan could complement or support this work (and to avoid replication).

Following partnership board meeting 3, the final draft of the action plan was emailed to all members of the co-design team and partnership board for final comments or suggestions. The research team also explored the recent literature on whole-school approaches to food to scope out relevant work being undertaken by other research groups to identify potential collaborators. Once all comments had been received, two members of the research team (WB and MB) updated the action plan, consolidating all ideas and discussions that had been provided by the co-design team, research team and partnership board up to that point. It was decided that the final set of actions would need to be conceived within two separate work packages to streamline activities: one to support schools directly in implementing key principles of the approach and the second to influence change within the wider system (e.g. policy level).

#### Co-design workshop 6

The aim of workshop 6 was to agree on how each action would be delivered within the two work packages. During the workshop, it was agreed that actions in work package 1 would be delivered via an online resource for schools, as this would be the most accessible and acceptable mode of delivery for schools. It was agreed that actions in work package 2 (wider systems changes) would be delivered by collaborating with other organisations (e.g. school food organisations, local government authority, academic institutions and the UK Government DfE) who were already advocating for systems-wide change.

### Step 6: Development of work packages 1 and 2

#### CONNECTS-Food resource (work package 1)

The CONNECTS-Food online resource was designed by members of the research team in collaboration with the co-design team and a website designer. Before the online resource was launched to the public, a draft was presented to the partnership board and academics in the field for review and feedback. The final resource (www.CONNECTS-Food.com) was made available to schools and promoted through press release and social media.

#### Development of impact and collaboration work package (work package 2)

The development of work package 2 was facilitated by members of the research team (MB and WB) who initiated a series of meetings with potential collaborators to develop a strategy for influencing change in the wider system. A dissemination plan was developed to promote the impact of the work, and further research was planned.

## Results

### Step 1: Understanding how the school food system operates

Our systems map has previously been published^(22)^, which enabled us to understand how the school food system operates (Additional File 2).

### Step 2: Convening the co-design team

Our co-design team is described in Table 2.

**Table 2.** Co-design team members

Stakeholder	*n*	Location of employment
Headteacher of a primary school	3	*n* 2 Yorkshire and Humber *n* 1 West Midlands
Local authority school food improvement officer	3	*n* 1 Yorkshire and Humber *n* 1 North East England *n* 1 Greater London
Lead from the external catering company	3	*n* 2 Scotland *n* 1 South West England
Representative from the national school food organisation	2	*n* 2 National
Community dietician	1	*n* 1 Greater London

### Step 3: Defining a whole-school approach to food (study aim 1)

We identified seven key principles of a whole-school approach to food: (1) The ‘priorities of headteachers and senior leadership team’, which include objectives aimed at encouraging headteachers to provide leadership in setting a positive food culture. (2) ‘Food on the curriculum’, which includes objectives that encourage headteachers and teachers to ensure that learning with and about food is incorporated into lessons. (3) ‘School food provision’, which includes objectives to encourage the headteacher and catering teams to improve the school food offer. (4) ‘School food policy and culture’, which includes objectives for headteachers and other stakeholders to ensure that children receive consistent messages about food, which is reflected in policy and overall culture. (5) The ‘dining experience’, which includes objectives to encourage the headteacher and catering teams to improve the lunchtime environment. (6) ‘Stakeholder engagement’, which includes objectives for senior leadership teams to actively engage with children, their families and the wider community in school food activities. (7) ‘Pastoral care’, which includes objectives for senior leadership teams to support families to access nutritious food. Our full list of whole-school approaches to food objectives is provided in Additional File 3.

### Step 4: Identifying leverage points within the school food system (study aim 2)

Leverage points identified through discussion with the co-design team are detailed in Table 3 and summarised below according to each key principle.

**Table 3.** Leverage points identified for each key principle

Key principle	Degree of leverage within overall system (according to Action Scales Model) (*highest to lowest; beliefs of system architects, system goals, system structures, system events)*	Leverage points identified by co-design team
Priorities of school leadership teams: ensuring that school leadership teams are committed to adopting a whole-school approach to food.	Goals	School expectations set out by the Department for Education (DfE) and the inspection framework used by the Office for Standards in Education, Children’s Services and Skills (Ofsted) are influential over whether headteachers and other members of the senior leadership team in schools would be willing to commit to adopting a whole-school approach to food.
	Structures	State schools, which sit within and are funded by local governments, require the support of their local government bodies to commit to adopt a whole-school approach to food.
	Events	School stakeholders require knowledge of how to deliver a whole-school approach, as well as confidence to adopt the approach.
	Structures	Stakeholders must perceive they have enough time, money and staff capacity to implement the approach.
Food in the curriculum: learning with and about food.	Goals	Expectations set by DfE and Ofsted are influential over whether schools would be willing to adopt changes to their wider curriculum to incorporate learning with and about food.
	Beliefs	Teachers need to be motivated to adopt changes to the curriculum and perceive that they have the relevant skills.
	Events	Relevant funding needs to be available to pay for recipe ingredients or to pay for the resources in which to cook (e.g. providing portable stoves and cooking equipment to be placed in classrooms).
School food provision: ensuring high-quality and appealing food is on offer to children.	Structures	The adoption of changes to school food provision may need to be instigated at the local authority level or an in-house catering team.
	Beliefs	Headteachers need to prioritise seeking out new and better contracts.
	Events	Catering team needs to have the relevant skills to adopt changes to the school food provision.
School food policy and culture: Ensuring school policies support whole-school approach to food ethos.	Beliefs	Headteachers, DfE and Ofsted need to prioritise the adoption of policies and initiatives that support a whole-school approach to food ethos.
	Beliefs	School stakeholders (e.g. teachers, pupils and parents) need to be supportive of the adoption of policies and initiatives that support a whole-school approach to food ethos.
	Structures	A high staff turnover makes it difficult to sustain the implementation of a whole-school approach to food policies, which influences decisions around whether to adopt them.
Dining experience: ensuring a pleasant eating environment for children.	Structures	Practical barriers influence whether a headteacher would choose to adopt changes in their school to ensure a pleasant dining experience for children. For example, a lack of time in the day to accommodate long lunch breaks, along with the availability of funds and physical space to facilitate changes to the dining area.
	Beliefs	The support of teachers and caterers is required to encourage headteachers to adopt changes to the dining experience.
Stakeholder engagement: involving a range of school stakeholders in the delivery of a whole-school approach to food.	Event	Schools (headteachers and other stakeholders) need to understand the benefits of engaging with stakeholders in order to adopt changes to their stakeholder engagement practices.
Pastoral care: supporting children and their families to access good food.	Structures	Challenges within school budgets could be a barrier to adopting this principle, for example, potential restrictions on the number of families that can access free school meals.
	Structures	Establishing a good relationship between families and the school is important for facilitating adoption of this principle so that potential issues to accessing healthy food can be identified.
	Structures	Knowledge levels among staff around eating behaviours of concern (e.g. children being hungry, eating disorders and poor diets) are influential over whether schools feel confident to support children and their families to access good food.

#### Priorities of school leadership teams

The expectations of schools as set out by the UK Government DfE and the inspection framework used by the Office for Standards in Education, Children’s Services and Skills (Ofsted) were perceived by the co-design team as being highly influential over whether headteachers and other members of the senior leadership team in schools would be willing to commit to adopting a whole-school approach to food (beliefs/goals). State schools, which sit within and are funded by local governments, were also perceived to require the support of their local government bodies (goals). Knowledge of how to deliver a whole-school approach to food was described as important (events), as was the headteacher’s confidence to adopt the approach. It was also recognised that the key to influencing adoption was alleviating concerns that the approach would require substantial time, money and staff capacity (structures).

#### Food in the curriculum

The expectations set by DfE and Ofsted were perceived by the co-design team to be highly influential over whether schools would be willing to adopt changes to their wider curriculum to incorporate learning with and about food (beliefs). As this is not currently mandatory within UK primary schools, the co-design team felt that teachers would need to be motivated to adopt the changes themselves and perceive that they have the relevant skills, training, confidence and budget (beliefs).

#### School food provision

Members of the co-design team explained that the decision to adopt changes to school food provision would sometimes be made at the local authority level but other times by an in-house catering team (structures). The priorities of the headteacher were again highlighted, who were perceived to have the power to seek out new and better contracts with caterers if they wanted to (beliefs), as well as the skills of the individuals within school catering teams (structures).

#### School food policy and culture

The extent to which the DfE and Ofsted prioritised a whole-school approach and the perceived beliefs and values of other school stakeholders (e.g. teachers, pupils and parents) was felt to be influential over whether headteachers would choose to adopt changes to policy and culture (beliefs). High staff turnover was mentioned as making it difficult to sustain the implementation of a whole-school approach to food policies, which could also influence decisions around whether to adopt new policies and initiatives (structures).

#### Dining experience

Some practical barriers were believed to influence whether a headteacher would choose to adopt changes in their school to ensure a pleasant dining experience for children. For example, a lack of time in the day to accommodate long lunch breaks, along with the availability of funds and physical space to facilitate changes to the dining area (structures). Gaining the support of teachers and caterers was also described as important (beliefs).

#### Stakeholder engagement

The co-design team explained that it was the role of the headteacher to instigate involvement of stakeholders from within and outside of the school. However, it was acknowledged that other stakeholders from within the school could also support the adoption of this principle by leading engagement with others. For example, caterers could engage with children regarding menu design, and teachers could involve parents in school events that incorporated food.

#### Pastoral care

The co-design team agreed that challenges within school budgets could be a barrier to adopting this principle, for example, potential restrictions on the number of families that can access free school meals (structures/goals). Establishing a good relationship between families and the school was described as important for facilitating the adoption of this principle so that potential issues in accessing healthy food could be identified (structures). Knowledge levels among staff around eating behaviours of concern (e.g. children being hungry) were also described as being influential over whether schools felt confident to support children and their families to access good food (structures).

Based on these discussions, a list of thirty-eight potential leverage points to influence within the action plan was produced, which was shortened to twenty during the scoring exercise (Table 4).

**Table 4.** CONNECTS-Food action plan

Key principle	Leverage point	Degree of leverage within overall system (according to Action Scales Model) (*highest to lowest; beliefs of system architects, system goals, system structures, system events)*	Action	Informed by (co-design team, partnership board or research team)	Delivery mode (work package 1 or 2)
Priorities of school leaders	Extent to which the whole-school approach to food is expected and monitored by Ofsted and the Department for Education	System goals	Collaborate with the Department for Education to develop a reporting template and statement builder for schools to display their whole-school approach to food statement on their website Collaborate with School Food Matters to develop a school food policy template for schools.	Partnership board	Work package 2
	Extent to which local authorities actively support a whole-school approach to food	System structure	Work with local authorities to set out school food strategies including signposting schools to the CONNECTS-Food resource	Partnership board	Work package 2
	Extent to which the senior leadership team understands or appreciates the potential impact of a whole-school approach to food on children, families and wider community.	Beliefs of system architects	Demonstrate the impact of whole-school approaches to food through the provision of evidence and case studies	Co-design team	Work package 1
	Level of confidence among the senior leadership team to implement a whole-school approach to food.	System event	Provide the senior leadership team with tools and guidance on how to implement a whole-school approach to food	Co-design team	Work package 1
Food on the curriculum	Priority and skills of teachers to develop their own food curriculum outside of the Ofsted framework	System events	Offer/signpost head teachers and teachers to relevant teaching resources and training	Co-design team	Work package 1
	Level of knowledge around health and safety considerations	System events	Offer/signpost head teachers and teachers to relevant teaching resources and training	Co-design team	Work package 1
	Presence of a food champion to lead on food curriculum	System structure	Encourage schools to enlist a food champion to lead on food curriculum	Co-design team	Work package 1
School food provision	Complexity of the procurement and contracting process	System structure	Provide guidance to schools on how to seek out and develop good catering contracts and/or use local and seasonal suppliers	Co-design team	Work package 1
	Extent to which catering teams receive training on how to make tasty and appealing foods	System structure	Persuade schools to upskill kitchen staff to ensure all food offered is healthy and tasty by signposting to training opportunities and case studies	Co-design team	Work package 1
	Extent to which parents support healthy food menus	Beliefs of system architects	Encourage schools to engage with parents and children to increase receptiveness to healthy food	Co-design team and partnership board	Work package 1
School food policy and culture	Extent to which parents and school governors support school food policies	Beliefs of system architects	Provide examples to schools of how they can successfully engage with families to promote support for a whole-school approach to food	Co-design team and partnership board	Work package 1
	Extent to which schools implement and monitor the performance of policies that support a whole-school approach to food	System goals	Support schools to develop and implement food policies including non-food rewards, dessert-free lunches, celebration food policy, removal of ‘Chip Friday’	Partnership board	Work package 1
	Extent to which lunchtime staff are involved in implementing healthy initiatives	System structure	Provide examples to schools of how lunchtime staff can be integrated into the wider school, for example, involving them in other areas of the school day such as lessons or parents’ evenings	Co-design team	Work package 1
Dining experience	Amount of time in the school day available to accommodate long lunch breaks	System structure	Persuade headteachers to extend lunch break by offering examples of how other schools have achieved this and demonstrating the evidence base, which supports the benefits	Co-design team	Work package 1
	Extent to which teachers and headteachers are willing to eat lunch in the dining hall	System structure	Persuade headteachers to request that teachers eat lunch in the dining by providing examples and demonstrating the evidence base, which supports the benefits	Co-design team	Work package 1
Stakeholder involvement	Extent to which the school council is utilised to support a whole-school approach to food	System structure	Encourage schools to engage with the school council through a whole-school approach to food process	Co-design team	Work package 1
	Extent to which schools are able to access school trips (e.g. proximity to local farms)	System event	Offer examples to schools on how other schools have organised school trips	Co-design team	Work package 1
Pastoral care	Ability of schools to make free school meals indistinguishable from paid school meals	System structure	Provide examples to schools of how others have implemented payment/dining systems so that school meals are not distinguishable from free school meal provision. FSM	Partnership board	Work package 1
			Explore the potential for autoenrollment of eligible families for free school meals	Research team	Work package 2
	Extent to which schools have a close relationship with parents to promote a supportive environment at home	System structure	Persuade schools to hold regular activities that involve parents to bring together school and families, for example, involving parents in delivering and attending cooking lessons	Co-design team	Work package 1
	Knowledge levels of staff on how to pick up on eating behaviours of concern	System structure	Provide training /signpost to training on identifying eating behaviours of concern and how to offer wider support to children and families	Co-design team	Work package 1

### Step 5: Development of the action plan (study aim 3)

The action plan is detailed in Table 4. In brief, actions to promote the commitment of headteachers to adopt a whole-school approach to food included collaborating with the DfE to monitor school-level adoption of the approach. Other actions included signposting headteachers and teachers to relevant training and resources to support them to adopt key principles, as well as providing examples of other schools that have successfully adopted the principles.

### Step 6: CONNECTS-Food action plan delivery

#### Work package 1 (online resource for schools)

The delivery of actions in this work package aimed to offer implementation support to schools as a mechanism to promote motivation, knowledge and confidence to adopt the approach via an online resource. The resource includes the provision of a self-review tool to help schools determine to what degree they are already delivering the approach, highlight areas for improvement and support them in developing their whole-school approach to food public statement (to be displayed on their website).

#### Work package 2 (impact and collaboration)

The delivery of actions within this work package is ongoing. To date, they have included being involved in an ongoing collaboration with DfE to support and monitor the adoption of whole-school approaches to food. Four local authorities in the UK have agreed to use the CONNECTS-Food resource to support their local evaluations of whole-school approach adoption. A research collaboration is underway with the Fix Our Food in Schools project^(27)^ in which the CONNECTS-Food key principles are used as a framework to measure the current adoption of a whole-school approach to food in schools in the UK.

## Discussion

This paper describes the co-design of a systems-wide action plan devised to promote the adoption of whole-school approaches to food. We defined key principles of the approach and considered how the school food system works towards or against the adoption of each principle. We applied the ASM^(15)^ to identify leverage points and develop our action plan, seeking guidance from our partnership board, consisting of regional and national school stakeholders. The resulting list of actions forms two work packages: one to support schools in implementing a whole-school approach to food and the other to influence change in the wider system.

Stage one of the ASM process involved developing an understanding of the school food system. Group model building is widely used to understand complex problems in public health, such as the causes of obesity^(28)^ and inadequate fruit and vegetable intake in children^(29)^, but there are few examples of using this as a starting point to design a systems-wide action plan. One such example, however, is the work of Pinzon *et al*.^(30)^, who used group model building and ASM guidance to develop a systems approach to tackle obesity-related behaviours in adolescents. In line with our systems-wide approach, their action plan included setting up collaborations with local authorities to improve the food environment as well as undertaking further research. Authors say that their actions were only the starting point for change, with measurable change only likely after several years, although through ongoing monitoring, they identified that the most sustainable actions were those that were incorporated into existing initiatives, such as working with local authorities who were already committed to updating local food policies^(31)^.

Our action plan predominantly aimed to influence the *beliefs* of headteachers along with *system goals* as set by the DfE and local authorities. Others have also identified these as priority areas for change. For example, Schliemann *et al*.^(32)^, who undertook a priority setting activity in the UK to understand where future research efforts need to focus to improve the school food system, highlighted the importance of securing the buy-in of headteachers and policymakers so that school food policies are valued and prioritised. In another priority setting study, Johnson *et al*.^(33)^ explored barriers and facilitators to changing the school food system in Australia, identifying the need for governments to lead on changing the school food agenda to promote change at the local level.

Our systems approach offers direct support to schools through tools and guidance. The need to offer this support is surprising given the volume of previous initiatives that have been designed to support a whole-school approach to food delivery (e.g. Food for Life^(34)^ and the School Food Plan)^(35)^. A systematic review undertaken in 2020^(36)^ also highlighted the need for a two-pronged approach to support compliance with school food policy, including direct support for schools alongside wider systems change.

### Strengths and limitations

Our systems-wide approach was developed in collaboration with school-level and national stakeholders who were geographically and organisationally diverse. Our co-design team defined a whole-school approach to food by identifying key principles, which our findings suggest are still not well understood. These key principles are already being used in research and by local authorities to measure the implementation of the approach and can be used by others.

We invited eighty-one school stakeholders to be part of our co-design team. Of these, only twelve opted to participate. However, we ensured that we achieved representation from a range of school stakeholders, including headteachers, school food improvement officers and catering leads, in order to provide a ‘bottom-up’ perspective^(37)^. We acknowledge, however, that our co-design team did not involve school children. A recognised challenge in developing a systems approach with children is striking a good balance between systems theory and participant engagement, although this challenge could have been alleviated by using tailored methods such as drawing, storytelling and discussion^(38)^. We also strived to engage parents within our co-design team, but uptake was low. This could have altered the direction of our action plan, as we know from our systems mapping work and the wider literature that family engagement is a key component of a whole-school approach to food implementation^(8)^. The expertise of our research team and partnership board may have unintentionally influenced the objectives and scope of the CONNECTS-Food action plan, for example, the ongoing commitment of DfE to monitor the implementation of a whole-school approach to food and the advocacy work of school food organisations on our partnership board. However, a strength of this paper is that we clearly set out our methods for developing our approach, which is known to be lacking in some co-design studies^(39)^.

In this project, co-design team members were asked to solve the complex problem of promoting the adoption of a whole-school approach to food by prioritising leverage points to influence and developing an action plan. Ideally, in co-design projects, the problem-solution cycle is iterative, and interventions are tested and then adapted accordingly^(40)^. The scope of the current study did not extend to monitoring the implementation and impact of our systems-wide approach to enable ongoing adaptation of our action plan, although future funding will be sought to explore these aspects. The method used to develop the CONNECTS-Food action plan involved drawing upon the experiences and expertise of our co-design team and partnership board, which was an iterative process. We chose to undertake meetings with our co-design team and partnership board separately; however, encouraging greater collaboration between the stakeholder groups could have supported the development of relationships between those stakeholders ‘working on the ground’ and those with greater input into national decision-making.

A limitation of the ASM framework is the lack of focused guidance on consolidating systems thinking with other disciplines such as implementation and behavioural science, which could strengthen the development of systems approaches by applying appropriate theory when considering leverage points and actions.

### Recommendations for future research

We recommend the use of co-design methods to develop systems approaches that address public health challenges such as poor adoption of initiatives that are known to be effective. Engaging implementation and beneficiary stakeholders ensures a good understanding and feasibility of leverage points and actions. Seeking engagement from key decision-makers including policymakers through collaboration, partnership and co-design has the power to support policy and environmental-level actions. Evaluation of systems-based studies is also strongly recommended, although not included in this study. Adequate time and resources are needed to monitor implementation and enable understanding of the impact and adaptation of actions. Assigning actions to smaller working groups with responsibility for delivery and regular reporting on progress and impact is key^(30,31)^. A comprehensive review of available systems frameworks is advised to identify which best fit the needs of the project, for example, use of relevant language, clear processes for transparent reporting and appropriate consideration to applicable theory and models from other disciplines (such as implementation science and behavioural theory) to promote successful outcomes.

### Conclusion

We identified that the beliefs and priorities of headteachers, along with the system’s goals set by the Department for Education, were the greatest leverage points for change in the UK primary school food system. Our co-design team was successful in supporting our understanding of how the school food system operates, identifying leverage points and agreeing upon actions. The methods described here can be replicated by others to understand leverage points for change within other school contexts and contribute to the growing literature on developing systems-wide approaches to support public health implementation.

## Supporting information

Burton et al. supplementary material 1 Burton et al. supplementary material

Burton et al. supplementary material 2 Burton et al. supplementary material

Burton et al. supplementary material 3 Burton et al. supplementary material

Burton et al. supplementary material 4 Burton et al. supplementary material

## Supplementary material

For supplementary material accompanying this paper, visit https://doi.org/10.1017/S1368980025101353.
