# Supplementary material for: Co-design of a systems-wide approach (CONNECTS-Food) to promote adoption of whole-school approaches to food
Source: Public Health Nutr. 2025 Oct 17;28(1):e188. doi: 10.1017/S1368980025101353 (PMC12722103; doi:10.1017/S1368980025101353)
Supplement: Burton et al. supplementary material 1 — Burton et al. supplementary material [file S1368980025101353sup001.docx]

| Workshop or meeting name | Aim and content |
| --- | --- |
| Partnership board meeting 1 | Aim: to provide input on the initial systems map of the school food system  Format: One hour Zoom meeting which involved the following steps   1. Partnership board members asked to introduce themselves and summarise their background etc. 2. A summary of the project to date was given, including details of the journey mapping and systems mapping workshops (step 1). 3. An image of the initial systems map was presented by a member of the research using the share screen function who talked through the map, including the four overarching domains and the nodes / factors within them. 4. The partnership board were invited to ask questions about the map and suggest if any nodes or connections were missing. 5. Notes were taken during the meeting, and the map was updated in response afterwards. |
| Co-design workshop 1 | Aims: 1) to provide feedback on the initial systems map, 2) to define the whole-school approach to food  Format: Two-hour workshop held on Zoom, involving the following steps   1. Co-design team members were introduced to one another and the co-design process and principles were explained   *Part 1: Providing feedback on the initial systems map*   1. An image of the initial systems map was presented using the share screen function. A member of the research team talked through the map, describing the four overarching domains and the nodes within them. 2. The co-design team members were encouraged to provide feedback on the map, including whether any nodes or connections were perceived to be missing.   *Part 2: Defining the whole-school approach to food*   1. Co-design team were given two minutes to think about what the whole-school approach to food means in practice and consider objectives of the approach which schools would be expected to adopt if implementing the approach. The team were also asked to name who would perform the objective (e.g. headteachers). 2. Team members were asked to enter considered objectives within the Zoom chat function. As team members entered objectives into the chat, the workshop facilitator read them out in turn and invited team members to discuss them in more detail. 3. During the discussion, objectives were written down by a member of the research team so that the list could be typed up afterwards. 4. After the workshop, the recording of the session was reviewed to ensure no objectives were missed. |
| Co-design workshop 2 | Aims: 1) to agree whole-school approach to food key principles  Format: Two-hour workshop held on Zoom, involving the following steps   1. A PowerPoint presentation was presented to the co-design team by a member of the research team using the share screen function. Each slide set out a whole-school approach to food key principle, including the objectives grouped within it. 2. As each key principle was presented, the workshop facilitator invited co-design team members to discuss the principle in more detail to help refine the concept and reach agreement among the team that it was representative of the approach. 3. After the workshop, a recording of the session was viewed with edits to the principles made in response. |
| Co-design workshop 3 | Aims: 1) to review the whole-school approach to food sub-systems maps 2) to identify potential leverage points to support adoption of whole-school approach to food key principles  Format: Two-hour workshop held on Zoom, involving the following steps  *Part 1: review of the whole school approach to food sub-systems maps*   1. An image of each sub-systems map was presented in turn using the share screen function. 2. As each map was presented, the facilitator asked the group: do you agree with the proposed factors influencing each principle? are any factors missing? are any factors more influential than others? 3. Notes were taken during the discussion to update the maps accordingly after the workshop.   *Part 2 identifying potential leverage points to support adoption of whole school approach to food key principles*   1. The co-design team were asked to consider potential leverage points within the school food system that could influence each sub-system to support adoption of whole-school approach to food principles. 2. As each key principle was considered in turn, the workshop facilitator read out a list of questions suggested by the Action Scales Model to guide identification of leverage points. Questions included: to what extent do policy makers prioritise this principle? (beliefs), what are the key goals (if any) that headteachers aim for which are related to this principle? (goals), how do physical structures influence adoption of this principle? (structures) and, what are the immediate barriers to adopting this principle? (events). 3. During the discussion, images of the sub-systems maps and the overall systems map were presented at appropriate time points using the share screen function to provide a visual point of reference. 4. During the discussions, a member of the research team listed the potential leverage points and reviewed a recording of the session afterwards. |
| Co-design workshop 4 | Aim: 1) to agree upon which leverage points to influence as part of CONNECTS-Food action plan  Format: Two-hour workshop held on Zoom, involving the following steps   1. Prior to the workshop all potential leverage points (as identified in workshop 3) were listed within a Google Form. At the start of the workshop, a link to the Google Form was shared with the co-design team. 2. Co-design team members were asked to draw upon their experience and expertise to assign each potential leverage point a score of 1-9 (1 being most feasible to influence and 9 being most challenging to influence). The co-design team were reminded that leverage points from each ASM weight category should be included in the action plan (as recommended by ASM guidance). Therefore, it was emphasised that even if a leverage point was perceived as being unfeasible to influence (e.g. *beliefs*), co-design team members should not to disregard these in the first instance, but instead to think closely about whether the CONNECTS-Food action plan could support the work of other organisations who may also be working towards influencing change in this area of the system (e.g., supporting work being undertaken by school food organisations based in the UK who are advocating for an overhaul of school food policy). 3. After the Google Form was completed by all team members, a generated summary of responses was presented back to the team using the share screen function. Where no consensus was reached on whether a leverage point was feasible to influence (i.e., no clear majority score between 1-9), reasons for disagreement were discussed among the team and a final decision was made on whether to include in the action plan. |
| Partnership board meeting 2 | Aim: 1) to generate initial ideas on how to influence agreed leverage points and begin to draft action plan  Format: One-hour meeting held on Zoom, involving the following steps   1. Prior to the meeting, an action plan template was developed in MS Excel, which was adapted from the ASM publicly available resources for developing a systems approach. 2. During the meeting, the action plan template was presented to the partnership board using the share screen function. Each leverage point was introduced in turn by the workshop facilitator and the partnership board members were asked to draw on their experience and expertise from a regional and national perspective to generate ideas on how they thought the leverage point could be influenced. 3. As the leverage points were discussed in turn, the action plan was populated accordingly by a member of the research team. |
| Co-design workshop 5 | Aim: 1) to build upon actions suggested by the partnership board to further develop the action plan  Format: Two-hour workshop held on Zoom, involving the following steps   1. The action plan template which was partially populated during Partnership board meeting 2 was presented to the co-design using the share screen function. Each leverage point was presented in turn, including the partnership’s board’s initial ideas on how to influence them. 2. As the action plan was being presented, co-design team members were asked to build upon the partnership board’s ideas, as well as to generate new ones, and the action plan was populated accordingly. 3. The co-design team were asked to consider whether they were aware of similar work being undertaken locally to avoid replication, or alternatively, whether CONNECT-Foods actions might support this work. |
| Partnership board meeting 3 | Aim: 1) to provide feedback and guidance on the action plan draft  Format: One-hour meeting held on Zoom, involving the following steps   1. The latest version of the action plan was presented by a member of the research team using the share screen function. 2. After the presentation, partnership board members were invited to provide feedback and further input. Notes were taken during the meeting so that the action plan could be updated accordingly afterwards. 3. Partnership board members were asked to describe if they were involved in or aware of other work aimed at promoting the adoption of whole-school approaches to food to understand whether the CONNECTS-Food action plan could complement or support this work (and to avoid direct replication). |
| Co-design workshop 6 | Aim: 1) to agree on how the action plan will be delivered  Format: Two-hour workshop held on Zoom, involving the following steps   1. A member of the research team gave a PowerPoint presentation which recapped on all previous stages of the co-design process. It was explained that the final stage in the process was to agree on how each action would be delivered within the two work packages. 2. The co-design team were split into two breakout rooms to discuss delivery of each work package in more detail, with one member of the research team facilitating the discussion in each room and one taking notes. Team members were given the choice of which breakout room they wanted to go in based on their own expertise and interest. 3. In each breakout room, the groups discussed each action in turn. |
