# Supplementary figures and images for: Co-design of a systems-wide approach (CONNECTS-Food) to promote adoption of whole-school approaches to food
Source: Public Health Nutr. 2025 Oct 17;28(1):e188. doi: 10.1017/S1368980025101353 (PMC12722103; doi:10.1017/S1368980025101353)

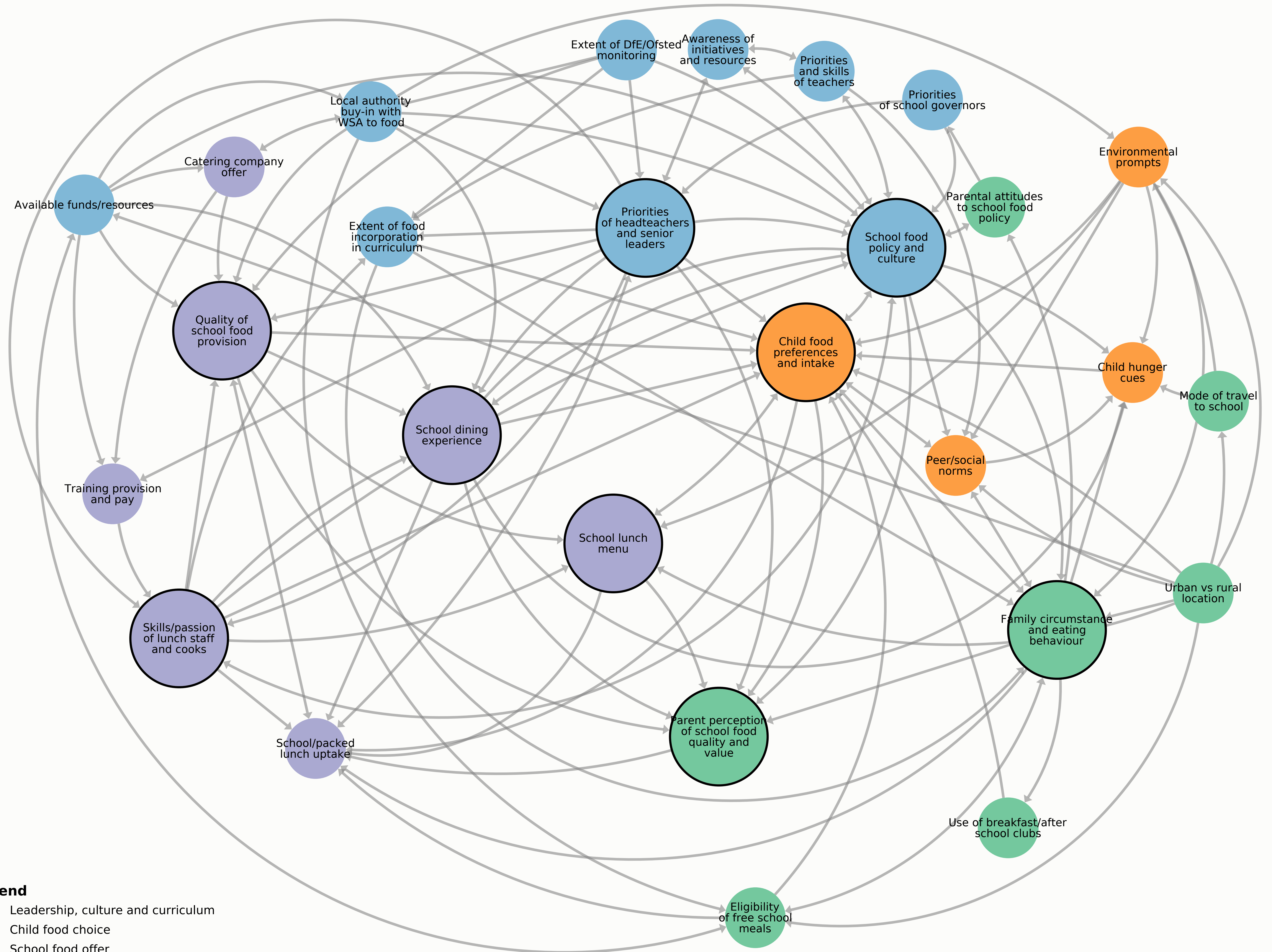

Supplement: Burton et al. supplementary material 2 — Burton et al. supplementary material [file S1368980025101353sup002.pdf]
