# Supplementary material for: Co-design of a systems-wide approach (CONNECTS-Food) to promote adoption of whole-school approaches to food
Source: Public Health Nutr. 2025 Oct 17;28(1):e188. doi: 10.1017/S1368980025101353 (PMC12722103; doi:10.1017/S1368980025101353)
Supplement: Burton et al. supplementary material 3 — Burton et al. supplementary material [file S1368980025101353sup003.docx]

Key principles of a whole school approach to food and objectives

| **Key principles of whole-school approach to food** | **Objectives** | **Identified by** | **Performed by** |
| --- | --- | --- | --- |
| Priorities of school leaders: Ensuring school leaders are committed to implementing a whole-school approach to food. | Provide leadership in setting a good food culture | Co-design team,  School Food Plan  School Food Top Marks | Headteachers, senior leaders and school governors |
|  | Monitor own performance of whole-school approach to food | School Food Top Marks |  |
|  | Use school as a hub for the local community | Co-design team |  |
|  | Ensure school cooks are acknowledged as school team members and include on school events (e.g., parents evening) | Co-design team,  School Food Plan |  |
|  | Ensure school contract is right, balancing high quality, appeal and cost | Co-design team,  School food plan |  |
|  | Make dining hall central to school, making food a vital element of school life | Food for Life |  |
| Food on the curriculum:  Learning with and about food | Ensure teaching is provided to children to improve food literacy | Co-design team  Food for Life | Teachers / headteachers |
|  | Use food as a way of bringing topics together across the curriculum | Co-design team,  School Food Plan |  |
|  | Allow continued professional development for teachers around learning with and about food | Co-design team  Food for Life |  |
|  | Provide funding / recompense teachers to pay for ingredients and cooking resource | Co-design team |  |
|  | Provide teaching with and about food beyond basic requirements | Co-design team |  |
|  | Ensure teaching is provided on sustainable school food systems | Co-design team |  |
|  | Grow food in school and use in school lunch | Co-design team,  School Food Plan  Food For Life |  |
|  | Arrange school trips to local farms and bakeries etc. | Co-design team  Food for Life |  |
| School food provision: Ensuring high quality and appealing food is on offer to children | Ensure a mix of familiar and new foods are available | Co-design team  School Food Plan | Catering teams and headteacher |
|  | Offer food samples to encourage children to try new food | Co-design team |  |
|  | Ensure meals comply with nutritional standards | Food for Life |  |
|  | Source food from sustainable and local food sources | School Food Plan  Food for Life |  |
|  | Engage local authority / academy leads to support improvement of school food offer | Co-design team |  |
|  | Introduce meat free lunches and more vegetable options | School Food Matters, healthy zones |  |
|  | Ensure catering teams are well trained and receive appropriate pay | Co-design team  Food for Life |  |
| School food policy and culture:  ensuring school policies support whole-school approach to food ethos. | Ensure children receive consistent messages about food reflected in policy and overall culture | Co-design team,  School Food Plan,  School Food Matters, healthy zones,  School Food Top Marks | Headteachers and other relevant stakeholders |
|  | Choose classroom rewards that are not sweets | Co-design team,  School Food Plan,  School Food Matters, healthy zones |  |
|  | Provide dessert free lunches | School Food Matters, healthy zones |  |
|  | Give children the opportunity to prepare or serve food | Co-design team |  |
|  | Ensure foods served at breakfast club and after school club are balanced and of high nutrient quality | Co-design team,  School Food Plan,  School Food Top Marks |  |
|  | Make tap water available at all times | School Food Plan,  School Food Matters, healthy zones  School Food Top Marks |  |
|  | Introduce packed lunch and break time policy and healthy incentives (in collaboration with parents and children) | Co-design team,  School Food Plan,  School Food Matters, healthy zones  Food for Life |  |
|  | Participate in national health initiatives | Co-design team |  |
|  | Hold food themes events | School Food Plan  Food for Life |  |
| Dining experience:  Ensuring a pleasant eating environment for children | Ensure lunch break allows time for eating and other activities | Co-design team,  School Food Plan, | Headteachers, teachers and catering teams |
|  | Ensure teachers eat in the dining hall | Co-design team |  |
|  | Ensure eating environment is pleasant for all, including younger children who may be intimidated by busy lunch environment | Co-design team,  School Food Plan,  School Food Top Marks |  |
|  | Keep queuing times short in the dining hall | School Food Plan |  |
|  | Make menus available online | Co-design team  School Food Plan |  |
|  | Replace prison-style trays | Co-design team,  School Food Plan  Food for Life |  |
|  | Ensure catering teams encourage children to experiment | Co-design team |  |
| Stakeholder engagement: Involving a range of school stakeholders in the implementation of a whole-school approach to food. | Engage with wider community to promote school activities | Co-design team,  School Food Plan  Food for Life | Senior leaders and other relevant stakeholders |
|  | Gain support of parents and governors in school policy decisions | Co-design team,  School Food Top Marks  Food for Life |  |
|  | Get the community involved in school food (e.g., grandparents and local chefs) | Co-design team,  School Food Plan  Food for Life |  |
|  | Involve children and parents in menu planning | Co-design team  School Food Plan |  |
|  | Offer after school cooking lessons for parents and children | Co-design team |  |
| Pastoral care:  Supporting children and their families to access good food. | Use food events to bring families and local communities together | Co-design team,  School Food Plan | Senior leaders and other relevant stakeholders |
|  | Have cashless system to eliminate free school meal stigma | School Food Plan |  |
|  | Offer training to staff on eating behaviours of concern and how to approach parents if needed | Co-design team |  |
|  | Offer free school meals and breakfast schemes for all | Co-design team |  |
|  | Ensure all free school meals eligible families are made aware of how to enrol, and offer support to enrol where needed | Co-design team |  |
|  | Offer lunch discounts / offers for siblings | School Food Plan |  |
|  | Consider ways in which children can be fed during school holidays | Co-design team |  |
