# Supplementary material for: Co-design of a systems-wide approach (CONNECTS-Food) to promote adoption of whole-school approaches to food
Source: Public Health Nutr. 2025 Oct 17;28(1):e188. doi: 10.1017/S1368980025101353 (PMC12722103; doi:10.1017/S1368980025101353)
Supplement: Burton et al. supplementary material 4 — Burton et al. supplementary material [file S1368980025101353sup004.docx]

| **Key principle** | **Leverage point scored as easier to influence** | **Leverage point scored as uncertain** | **Leverage point scored as harder to influence** |
| --- | --- | --- | --- |
| Priorities of school leaders | Extent to which school leaders are able to navigate whole school approach to food initiatives and guidelines (event) | Extent to which local authorities actively support whole school approach to food (beliefs / goals) | Extent to which schools are required to implement whole school approach to food as set out by DfE and Ofsted (beliefs / goals) |
|  |  | Extent to which senior leaders understand or appreciate the potential impact of a whole school approach to food on children, families and wider community (beliefs) | Turnover of staff that are instrumental in implementing a whole school approach to food (structures) |
|  |  | Level of confidence senior leaders have to implement whole school approach to food (event) | Availability of funds, resources and capacity required to make necessary changes (structures) |
| School food provision | Level of understanding among catering teams on school food standards and guidelines (event) | Level of school food meal uptake which can drive costs up or down (structures) | Extent to which headteachers prioritise improving the school food provision (beliefs) |
|  |  | Receptibility of children to try new foods presented to them (beliefs) | Extent to which parents support healthy food menus (beliefs) |
|  |  | Costs of preparing healthy food vs unhealthier foods (structures) | Availability of funds and space needed to prepare fresh food (structures) |
|  |  | Extent to which catering teams receive training on how to make tasty and appealing foods and are paid fair salaries that make them feel valued (structures) |  |
|  |  | Complexity of procurement and contracting process (structures) |  |
| Food on the curriculum | Level of knowledge around health and safety considerations (event) | Priority and skills of teachers to develop own food curriculum outside of Ofsted framework (beliefs) | Extent to which schools are required by DfE and Ofsted to incorporate food into the wider curriculum (beliefs / goals) |
|  | Presence of food champion to lead on food curriculum (structure) | Availability of funds and space for resources (structures) |  |
|  |  | Extent to which other subjects on the curriculum are prioritised over food topics (goals) |  |
| School food policy and culture | None | Extent to which schools implement and monitor performance of policies that support whole school approach to food (goals) | Extent to which headteacher prioritises implementation of school food policy and a positive food culture (beliefs) |
|  |  |  | Influence of food retailers offering unhealthy foods in the wider school food environment (structure) |
|  |  | Level to which lunch time staff are involved in implementing healthy initiatives (structures) | Influence of marketing and advertising in the media (structures) |
|  |  |  | Extent to which parents and school governors support for school food policies (beliefs) |
|  |  |  | Turnover of staff that are instrumental in implementing school food policies (structures) |
| Dining experience |  | Amount of time in the school day available to accommodate long lunch breaks (structures) | Availability of funds, time and physical space to facilitate change to eating environment (structures) |
|  |  | Extent to which teachers and headteachers are willing to eat lunch in the dining hall (beliefs) | Priority of headteachers and local authorities to prioritise making improvements to the dining experience (beliefs) |
|  |  | Extent to which catering staff are embedded throughout the whole of the school (structures) |  |
| Stakeholder engagement | Presence of dedicated champion to lead on stakeholder engagement (e.g., teachers seeking out school trips) (structures) | Extent to which schools are able to access school trips (e.g., proximity to local farms) (structures) | Priorities of headteachers to lead on stakeholder engagement (beliefs) |
|  | Extent to which school council are utilised to support whole school approach to food (structures) | Extent to which stakeholders understand the benefits of wider stakeholder engagement (event) |  |
| Pastoral care | Ability of school to make free school melas indistinguishable from paid school meals (structures) | Level to which school leadership understand / appreciate how a whole school approach to food fits within wider social context | Level to which children can access free school meals (e.g., extending free school meals eligibility) (structures / goals) |
|  | Knowledge levels of staff on how to pick up on eating behaviours of concern (structures) |  |  |
|  | Ensure all school children that are eligible for free school meals are enrolled (structures) |  |  |
